# Endoscopic septotomy of rectal transmural septum perpetuating a rectal leak

**DOI:** 10.1055/a-2587-8969

**Published:** 2025-05-06

**Authors:** Pedro Marílio Cardoso, Guilherme Macedo, Eduardo Rodrigues-Pinto

**Affiliations:** 1 285211 Gastroenterology Department, Centro Hospitalar Universitário de São João, Porto, Portugal; 2 26705 Faculty of Medicine, University of Porto, Porto, Portugal

A 58-year-old woman with a past medical history of rectocele underwent a Stapled Transanal Resection of the Rectum (STARR) procedure, which was complicated by a staple line leak. After a protective ileostomy, endoscopy revealed a leak involving half of the luminal circumference, with a 9 cm long associated collection ( Fig. 1 ). Three sessions of intracavitary endoscopic vacuum therapy were performed, including two simultaneous foams (EndoSponge, Boston Scientific) ( Fig. 2 ). Despite the improvement of endoscopic findings, leak and collection dimensions persisted, with the development of a proximal communication between the collection and the rectal lumen, configurating a double lumen, due to the presence of a transmural wall septum between the collection and the rectum ( Fig. 3 ). Endoscopic septotomy of the transmural septum was then performed with a grasping-type scissor (ClutchCutter, Fujifilm) ( Video 1 , Fig. 4 ). Additional hotsnare polipectomy of the cut septum was performed, with the placement of two detachable snares on each edge of the residual septum to prevent delayed bleeding. The patient was discharged 1 week later with improved clinical status. The bowel was reconstructed 3 months later. Endoscopy evaluation 6 months later revealed a completed re-epithelialization of the collection with leak resolution ( Fig. 5 ).

**Fig. 1:**
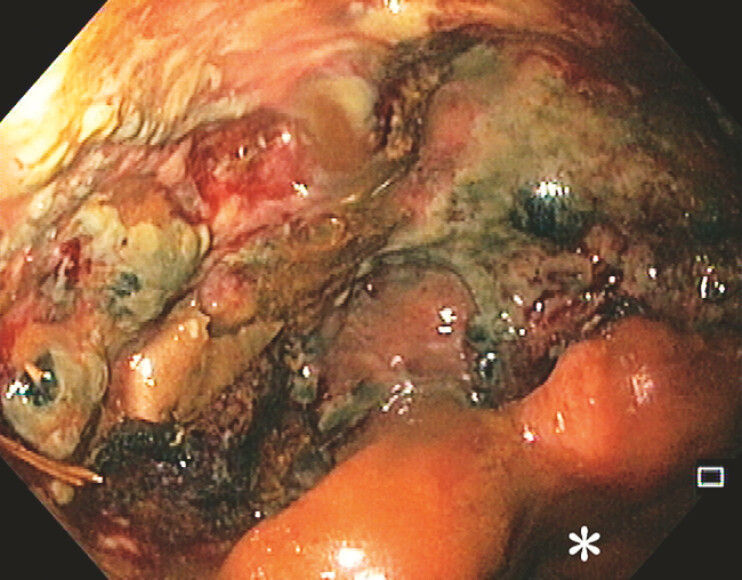
Leak involving half of the luminal circumference; *rectal lumen.

**Fig. 2:**
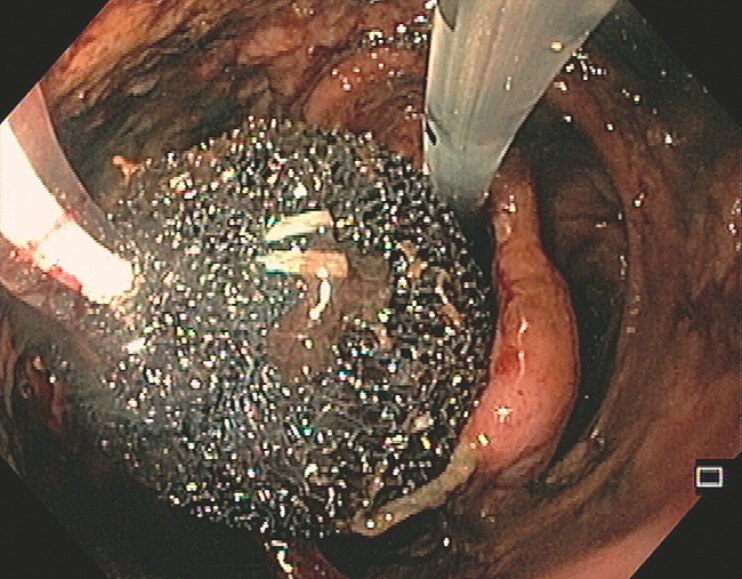
Vacuum therapy with two intracavitary sponges.

**Fig. 3:**
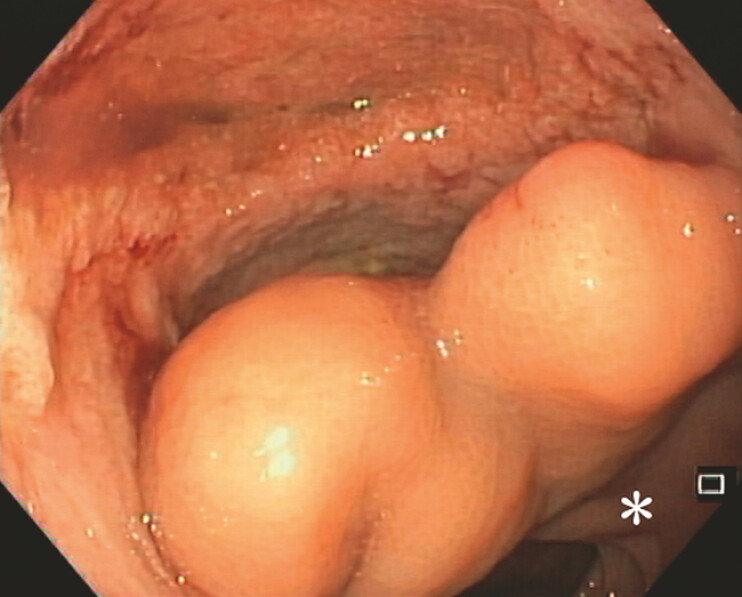
Endoscopic improvement of leak cavity with granulation tissue; *rectal lumen.

**Fig. 4:**
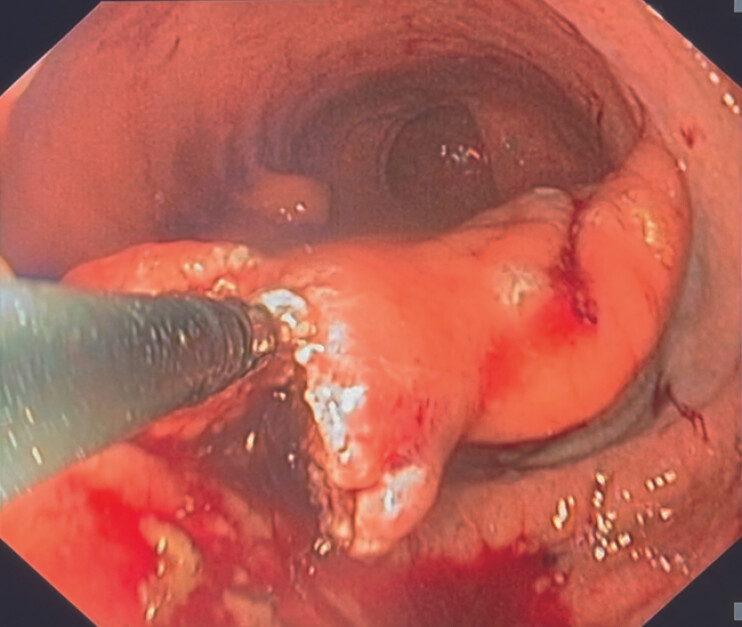
Endoscopic septotomy of the transmural septum.

**Fig. 5:**
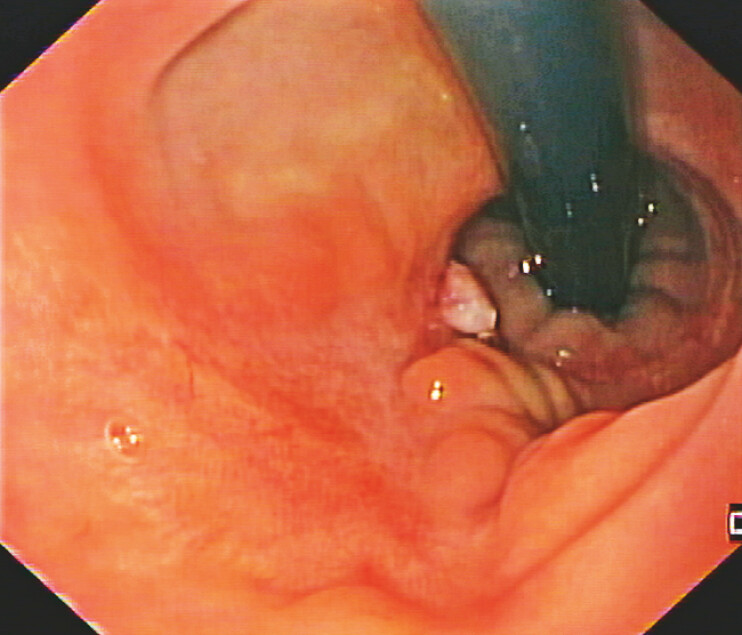
Retroflexion endoscopic view showing completed re-epithelialization of the collection and leak resolution.

Endoscopic septotomy of rectal transmural septum perpetuating a rectal leak.Video 1

Treatment of gastrointestinal leaks can be challenging, frequently requiring a multimodality approach, often during a prolonged period of time 1 . Improvement of leak drainage by equalization of the pressures between the rectum and the collection, similar to endoscopic septotomy in chronic gastric sleeve leaks 2 , may allow a faster resolution of the collection. To the best of our knowledge, this is the first report of a lower tract septotomy.

Endoscopy_UCTN_Code_CPL_1AJ_2AG

## Notes

Endoscopy E-Videos https://eref.thieme.de/e-videos
*E-Videos* is an open access online section of the journal *Endoscopy* , reporting on interesting cases and new techniques in gastroenterological endoscopy. All papers include a high-quality video and are published with a Creative Commons CC-BY license. Endoscopy E-Videos qualify for HINARI discounts and waivers and eligibility is automatically checked during the submission process. We grant 100% waivers to articles whose corresponding authors are based in Group A countries and 50% waivers to those who are based in Group B countries as classified by Research4Life (see: https://www.research4life.org/access/eligibility/ ). This section has its own submission website at https://mc.manuscriptcentral.com/e-videos .
